# Intersectional inequalities in younger women’s experiences of physical intimate partner violence across communities in Bangladesh

**DOI:** 10.1186/s12939-021-01587-z

**Published:** 2022-01-12

**Authors:** Laila Rahman, Janice Du Mont, Patricia O’Campo, Gillian Einstein

**Affiliations:** 1 Dalla Lana School of Public Health, University of Toronto, 155 College St., Suite- 620, Toronto, ON M5T 3M7 Canada; 2 Women’s College Research Institute, Women’s College Hospital, 76 Grenville St., 6th Floor, Toronto, ON M5S 1B2 Canada; 3 Centre for Urban Health Solutions, St. Michael’s Hospital, 30 Bond St., Toronto, ON M5B 1W8 Canada; 4 Li Ka Shing Knowledge Institute, St. Michael’s Hospital, 209 Victoria St., Toronto, ON M5B 1T8 Canada; 5 Department of Psychology, University of Toronto, 100 St. George St., Toronto, ON M5S 3G3 Canada; 6 Department of Gender Studies, Linköping University, 581 83 Linköping, Sweden

**Keywords:** Bangladesh, Communities, Domestic violence, Intercategorical intersectionality, Cross-sectional survey research, Young women

## Abstract

**Background:**

Physical intimate partner violence (IPV) risk looms large for younger women in Bangladesh. We are, however, yet to know the association between their intersectional social locations and IPV across communities. Drawing on intersectionality theory’s tenet that interacting systems of power, oppressions, and privileges work together, we hypothesized that (1) *younger, lower educated* or *poor* women’s physical IPV experiences will be exacerbated in disadvantaged communities; and conversely, (2) *younger, higher educated* or *nonpoor* women’s physical IPV experiences will be ameliorated in advantaged communities.

**Methods:**

We applied intercategorical intersectionality analyses using multilevel logistic regression models in 15,421 currently married women across 911 communities from a national, cross-sectional survey in 2015. To test the hypotheses, women’s probabilities of currently experiencing physical IPV among intersectional social groups were compared. These comparisons were made, at first, within each type of disadvantaged (e.g., younger or poor) and advantaged (e.g., older or nonpoor) communities; and then, between different types of communities.

**Results:**

While our specific hypotheses were not supported, we found significant within community differences, suggesting that *younger, lower educated or poor* women were bearing the brunt of IPV in almost every community (probabilities ranged from 34.0–37.1%). *Younger, poor* compared to *older, nonpoor* women had significantly higher IPV probabilities (the minimum difference = 12.7, 95% CI, 2.8, 22.6) in all communities. Similar trend was observed between *younger, lower educated* compared to *older, higher educated* women in all except communities that were poor. Interestingly, younger women’s advantage of higher education and material resources compared to their lower educated or poor counterparts was observed only in advantaged communities. However, these within community differences did not vary between disadvantaged and advantaged communities (difference-in-differences ranged from − 0.9%, (95% CI, − 8.5, 6.7) to − 8.6%, (95% CI, − 17.6, 0.5).

**Conclusions:**

Using intersectionality theory made visible the IPV precarity of younger, lower educated or poor women across communities. Future research might examine the structures and processes that put them at these precarious locations to ameliorate their socio-economic-educational inequalities and reduce IPV in all communities. For testing hypotheses using intersectionality theory, this study might advance scholarship on physical IPV in Bangladesh and quantitative intersectionality globally.

**Supplementary Information:**

The online version contains supplementary material available at 10.1186/s12939-021-01587-z.

## Introduction

Physical intimate partner violence (IPV) is a serious human rights and public health concern worldwide [1] and particularly in Bangladesh [2]. Although physical IPV risk looms large for all Bangladeshi women through their life courses, younger women are at a higher risk [3, 4], so are the women who are lower educated or poor [5–8]. However, as Bangladeshi IPV literature mostly focuses on women’s single-axis categories such as age, education, and poverty, we do not know what happens when these marginalized categories (hereafter referred to as *social locations*) intersect across different types of communities. Intersectionality theory [9–11], originated in Black feminism, invites us to go beyond women’s single-axis locations to examine the association between younger women’s intersectional social locations (which are socially produced sites of power, oppressions, and privileges) to add to our understanding of their heightened physical IPV risk. Although intersectionality has emerged as a critical feminist theory [12]; quantitative IPV literature is yet to apply and extensively engage with this theory. Also, of high importance is to know the role of communities in which women live. Although a review of contextual studies [13] suggested communities including poor communities affect IPV, we do not know how younger women’s intersectional locations interact with communities. In this article, we, therefore, have applied intersectionality theory to examine Bangladeshi currently married younger women’s intersectional social locations of education and poverty across different types of communities that shape their current experiences of physical IPV.

Using an intersectional analysis, a recent study has shown that younger, lower educated women had higher probabilities of experiencing physical IPV than older, higher educated women [14]. Although poverty is an important risk factor [4, 15] and communities in which women live affect their experiences of physical IPV [13, 16], this study did neither examine this outcome at women’s intersecting social locations of younger age and poverty, nor did it examine how these intersectional locations vary across communities. Since mid-1990s, studies have underscored the importance of communities in shaping women’s IPV in the United States [16] and around the world [13] including Bangladesh [17, 18]. A few studies [19, 20] have also examined interactions among women’s community- and individual-level single-axis locations (e.g., interaction between individual- and community-level income and women’s status) but not their intersectional locations. In Bangladesh, considering the role of child marriage and poverty [18, 21, 22], driven by patriarchy and neo-classical economy, disadvantaged communities in which high proportions of younger married and poor women live (hereafter referred to as *younger communities* and *poor communities*) are of particular interest.

Younger communities have likely been shaped by patriarchy–the underlying structure and ideology of men’s domination or power over women [23], which contributes to early marriage in Bangladesh [24]. In such communities, social norms encourage men to use violence to keep the men-dominated social order; therefore, public disapproval of women’s breaking social norms (such as taking jobs outside the home) may trigger IPV [3]. Thus, feminist theories suggest that women have greater IPV risk if they live in patriarchal younger communities [18]. Women, of course, adopt different strategies to escape violence [25, 26].

Due to macro social-economic policies and neoclassical economy, poor communities are likely shaped by scarcity of resources, infrastructure, and dearth of economic opportunities. In these communities, lack of income opportunities and resource constraints may trigger stress, consequently physical IPV [27]. They may also unable to provide social support and networks for women to resist and men to stop perpetrating violence [28]. In Bangladesh, a study [20] examining the effect of community-level factors found that community average per capita household income was a protective factor for physical or sexual IPV, although that association was marginally significant, while another study [17] examined but did not find association between community-level poverty and physical IPV, rendering the association between community-level poverty and IPV inconclusive. Given the lack of empirical evidence using nationally representative data, we wanted to examine whether the interaction between women’s intersectional social locations and community types affect their experiences of physical IPV.

### Conceptual framework

We used Crenshaw’s intersectionality theory [10, 11] and McCall’s intercategorical intersectionality approach [12] to conceptualize this study. In introducing intersectionality theory, Crenshaw [10] made visible Black women who were located at the intersection of two marginalized social locations: race and gender. Centring the experiences of Black women and Black, Indigenous, and people of colours (BIPOC), this theory highlights how individual social locations are produced and shaped by interacting and mutually constituting social structures and processes such as ageism, classism, sexism, and racism, exerting power over individuals to shape their experiences at different intersections of age, class, gender, and race, among others [29–31].

To advance this field of inquiry using quantitative methods, McCall [12] proposed the intercategorical approach, which quantifies the differences among different intersectional locations. Consequently, the field of quantitative intersectionality has continued to grow across disciplines including public health [32]. Encouraging as these applications are, a recent systematic review [33] revealed that exploratory analyses dominate intersectionality research. Although such studies are important, it is necessary to formulate and test theory-driven hypotheses that might help program managers/policymakers to understand and change the social structures to improve population health [33, 34]. Interestingly, intersectionality as a critical normative theory cannot be tested [35]. If thought of as an empirical paradigm [36] it is possible to select categories for research with “the knowledge of the research topic” ( [31] p1715).

**Fig. 1:**
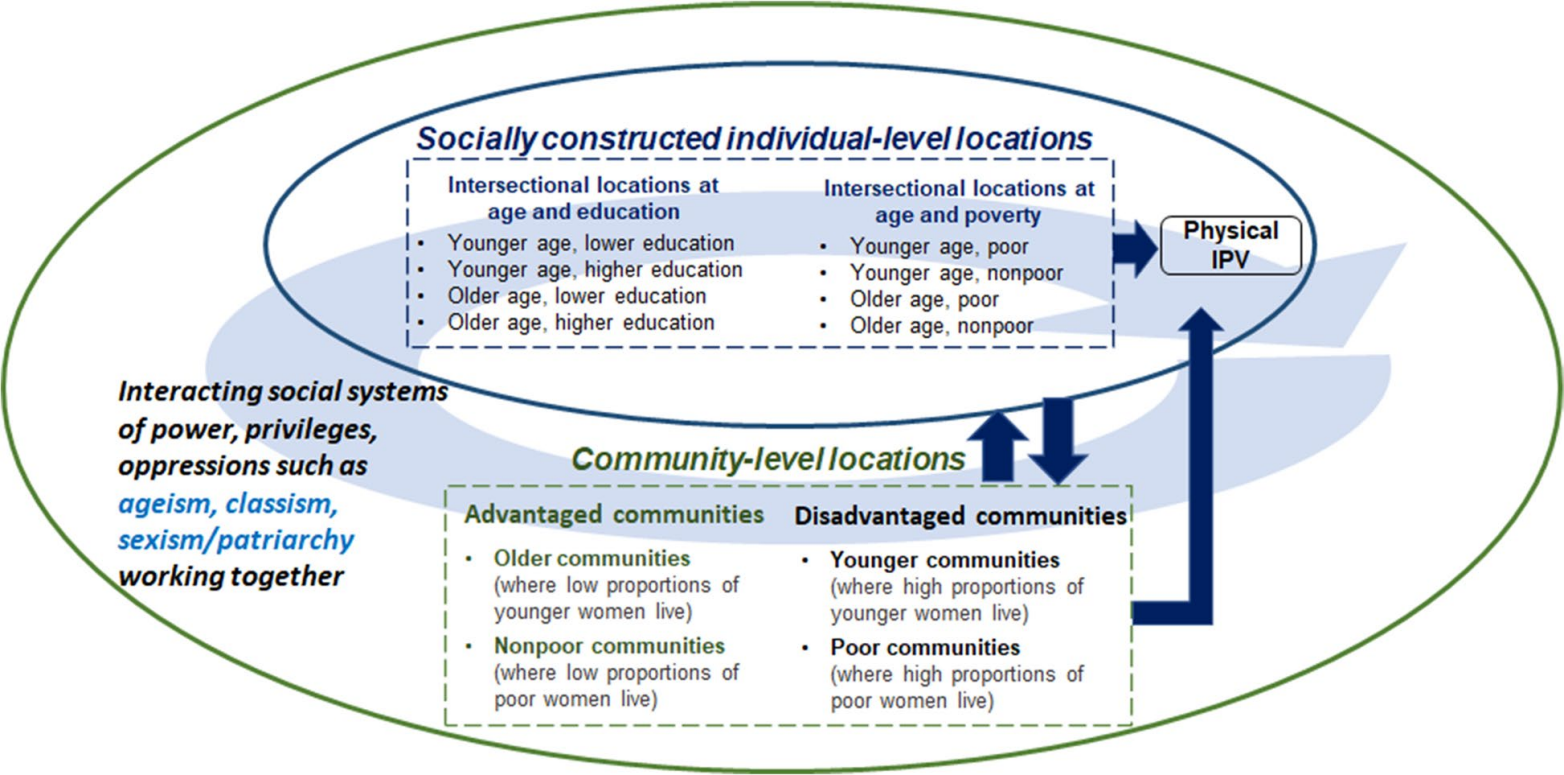
Conceptual model depicting women’s intersecting locations interacting with communities, shaping their physical intimate partner violence (IPV) experiences

We, therefore, drew on Bangladeshi IPV literature and intersectionality theory’s tenet that the interacting systems of power, oppressions, and privileges work together [30, 31] to generate hypotheses. We conceptualized that women’s individual-level intersecting locations interact with communities to shape their physical IPV experiences (Fig. 1). We hypothesized that Bangladeshi younger women who are lower educated or poor are more oppressed in disadvantaged communities (e.g., younger or poor communities) than their counterparts in advantaged communities (e.g., older or nonpoor communities). Interacting systems of power and privileges, on the other hand, might help younger women who are higher educated or nonpoor to be more successful in escaping physical IPV in advantaged compared to disadvantaged communities.

### Research questions and hypotheses

Given the above theoretical frame, we asked: (a) do *younger, lower educated* or *poor* women’s physical IPV experiences get exacerbated in disadvantaged communities? Conversely, (b) do *younger, higher educated* or *nonpoor* women’s physical IPV experiences get ameliorated in advantaged communities?

To answer the above questions, we tested two directional hypotheses: (1) Younger women who are lower educated or poor will have higher probabilities of experiencing physical IPV than older women who are higher educated or nonpoor; these differences in probabilities will be higher in disadvantaged than advantaged communities; and (2) Younger women who are higher educated or nonpoor will have lower probabilities of experiencing physical IPV than their counterparts who are lower educated or poor; these differences will be higher in advantaged than disadvantaged communities.

## Methods

### Study design

We employed an intercategorical intersectionality, multilevel, cross-sectional study design to test directional hypotheses [12, 37]. We used the latest nationally representative, secondary, cross-sectional survey data administered by Bangladesh Bureau of Statistics (BBS) that used a stratified two-stage cluster sampling design from the 2015 Bangladesh violence against women survey (BVAWS2015) following the World Health Organization and United Nations guidelines [38]. BBS considered design effect in calculating sample, selected households, and participants randomly, and trained interviewers to collect data, ensuring the privacy and confidentiality of women using a standard pilot-tested questionnaire.

### Study setting and participants

BBS [38] conducted BVAWS2015 survey in all the seven Bangladeshi administrative divisions, which were stratified by rural, urban, and city locations. Women 15-years and older living in the same household were considered eligible; however, guests and domestic workers who lived there for less than 6 months were excluded [38]. Woman interviewers recruited participants from their households across 911 primary sampling units (PSUs) which were geographical units with an average 108 households. After identifying households during 13–22 August 2015, woman interviewers conducted interviews in person with women and girls over a period of 15 days [38]. Only one participant per household was interviewed following the World Health Organization’s ethical guideline [38, 39].

BVAWS2015 interviewed 21,688 women who were 15 years or older [38]. In the current study, after excluding never married (*n* = 1701), previously married (*n* = 1799), women who were not living with a spouse during the survey (*n* = 2676), transgender (*n* = 76), unknown gender (*n* = 1), and women with missing information on outcome variable (*n* = 14), we included 15,421 participants in our final sample. These currently married 15 years or older women were living with their spouses during the survey across 911 communities (see Additional file 1).

We categorized PSUs as communities [7, 40]. These communities comprised four types: younger communities, poor communities, older communities, and nonpoor communities. For this categorization, we used ever-married women’s (*n* = 19,987) age and poverty characteristics. Each community type included at least 120 communities with an average 17 observations (minimum 8, maximum 25) per community. Also, 98.5% of communities had more than 10 observations. Considering the minimum requirement for community-level samples is 50 to 100 and individual-level samples for a 2-level model is 5 to 10 [41], we considered our community- and individual-level samples adequate.

As BVAWS2015 [38] had only 4.8% non-response rate and our sub-sample had negligible number of missing cases, the current study was able to reduce the non-response bias. BBS [38] also took measures to reduce the measurement bias by considering the design effect and selection bias by using a nationally representative sample and randomly selecting PSUs, households, and women [42, 43]. The interviewer training in asking sensitive questions that followed the ethical guideline [39] might have also helped reducing the interviewer and social desirability biases.

### Measures

The binary dependent variable–IPV, was constructed from the modified conflict tactics scale-2 [44], which is considered a gold standard measure [1]. Items that BBS [38] used to measure physically violent acts that women reported to have experienced in the past year included their getting: (a) slapped, punched or something thrown at them; (b) pushed, shoved or pulling of their hair; (c) burned with hot things; (d) acid thrown at them; (e) hot water/oil/milk/peas thrown at them; (f) kicked, dragged or beaten up; (g) suffocated or strangled; (h) burned; (i) threatened with knife/weapon; and (j) hit by a stick. Items c, d, e, and j were country specific items. We generated IPV by coding it 1 if a woman responded yes to any of these acts and, 0, if not.

Individual-level independent variables included younger age, lower education, and poor. We used binary coding to manage complexity and coding decisions were made using previous literature examples [4, 7, 14, 45]. Younger age was coded 1, if women were 15 to 29 years old; and 0 if they were 30 years and older. Lower education was coded 1, if women completed 0 to 4th grades; and 0, if they completed 5th grade or higher levels of education. BBS [38] generated wealth quintiles using household asset items to create five wealth groups. Using this, the poor category was coded 1, if a woman’s household belonged to 1st wealth quintile; and 0, if otherwise.

Community-level independent variables included two binary variables: (a) younger communities and (b) poor communities. We generated these community types by calculating proportions of ever-married younger women and poor women living in each community. Communities in which high proportions of younger women lived were coded as younger communities and the remainder as older communities. Similarly, communities where high proportions of poor women lived were coded as poor communities, and the remainder, as nonpoor communities. Thus, we distinguished four types of communities. High proportion cut-off points were determined based on the communities in which proportions of women with a specific characteristic (i.e., younger, poor) scored higher than one standard deviation (SD) above the mean. (See these cut-off points in Results section.) Using standardization addressed the rightly skewed poor community variable. This approach is consistent with earlier research [17, 46].

For covariates, we included dummy coded women’s religion (Islam = 1, Other = 0), geographical location (Rural = 1, Urban/City locations = 0), and their husband’s age (< 30 = 1, > = 30 = 0) and education (<5th grade = 1, > = 5th grade = 0). We treated them as confounders, because they are potentially associated with married women’s age, education, and poverty; and are also risk factors for IPV against women in Bangladesh [4, 19, 47–49]. We did not use women’s income variable as a confounder, as it potentially lies in the causal pathway between the dependent and independent variables.

### Statistical analyses

We carried out univariate analyses to describe the variables, bi-variate analyses to examine the distribution of independent variables by outcome, and logistic regression analyses for hypothesis testing. We conducted complete case analyses to address the missing data. We ran a two-level fixed effect logistic regression model with cross-product terms of explanatory main effects variables to predict IPV [37]. This model included women’s individual-level variables (i.e., younger age, lower education, and poor) and community-level variables (i.e., younger communities and poor communities; see Additional file 2). Relevant interaction terms for individual main effects and cross-level interaction terms between individual- and community-level variables were included following the well-formulated hierarchical design [50]. We adjusted for confounding variables and survey weights including sampling strata, clusters at the community-level, and households’ and women’s weights at the individual-level to avoid making types I and II errors [51].

For testing the discriminatory accuracy, we measured the intraclass correlation coefficient and incremental percentage change in the area under the receiver operating characteristic curve (AUC) between the null model (i.e., a benchmark model that included only the community identification variable) and the explanatory model [52, 53]; and also estimated the significant difference in AUC between these models. To estimate the measures of association, we calculated marginal predicted probabilities and significant differences across intersecting social locations, with pairwise comparisons and Bonferroni correction. We conducted sensitivity analyses with continuous instead of categorical level-1 variables; and divided communities into deciles (i.e., 10 equal population groups) by community-level mean age and mean wealth quintile values instead of using dichotomization (see Additional file 3).

We used 5% significance level in all estimates. Analyses were conducted in Stata version 15.1 [54]; and Stata’s [55] *margins* with *pwcompare* was used to calculate differences between women’s probabilities of experiencing IPV at different intersectional locations within communities; and *lincom* to measure the difference-in-differences between disadvantaged and advantaged communities. We used the strengthening the reporting of observational studies in epidemiology (STROBE) cross-sectional guidelines [56] to report this study.

## Results

### Women and community characteristics

Over one-third (34.0%, weighted n or n_w_ = 11,732,707) women were 15 to 29 years old; almost 50% (n_w_ = 17,108,771) had no or less than a fifth-grade education; and 23% (n_w_ = 7,944,569) were poor (see Additional file 4). At the intersection of age and education, 11.7% (n_w_ = 4,053,377) women were *younger, lower educated*, 22.3% (n_w_ = 7,679,330) were *younger, higher educated*, while 28.2% (n_w_ = 9,758,881) were *older, higher educated*. At the intersection of age and poverty, 7.9% (n_w_ = 2,735,813) women were *younger, poor*, 26.0% (n_w_ = 8,996,895) were *younger, nonpoor*, while 51.0% (n_w_ = 17,605,518) were *older, nonpoor*.

Over 82% (n_w_ = 28,387,282) women lived in advantaged communities (in which high proportions of older or nonpoor women lived) while at least 17.1% (n_w_ = 5,896,066) women lived in disadvantaged communities (in which high proportions of younger or poor women lived; see Additional file 4). High proportion cut-off points for defining community types were 43.3 and 41.8% for younger and poor communities, respectively. That is, in each younger community, at least 43.3% of the ever-married women were younger; and in each poor community, at least 41.8% of them were poor.

### IPV prevalence rates across women’s single-axis locations and types of communities

Overall, although 25.1% (n_w_ = 34,546,982) women reported experiencing IPV in the past year, the prevalence rates for younger, lower educated, and poor women compared to older, higher educated, and nonpoor women, respectively, were significantly higher (27.8–31.4%, n_w_ = 17,108,771–7,944,569 vs. 22.3–23.3%, n_w_ = 17,438,211–22,814,274; see Additional file 5). IPV prevalence rate did not vary between younger and older communities (25.7%, n_w_ = 6,159,700 and 24.9%, n_w_ = 28,387,282), but it did vary for women in poor compared to nonpoor communities (29.5%, n_w_ = 5,896,066 vs. 24.2%, n_w_ = 28,650,916, F (1, 890) =7.2, *p* = .01).

### IPV prevalence rates across women’s intersectional locations and types of communities

**Fig. 2:**
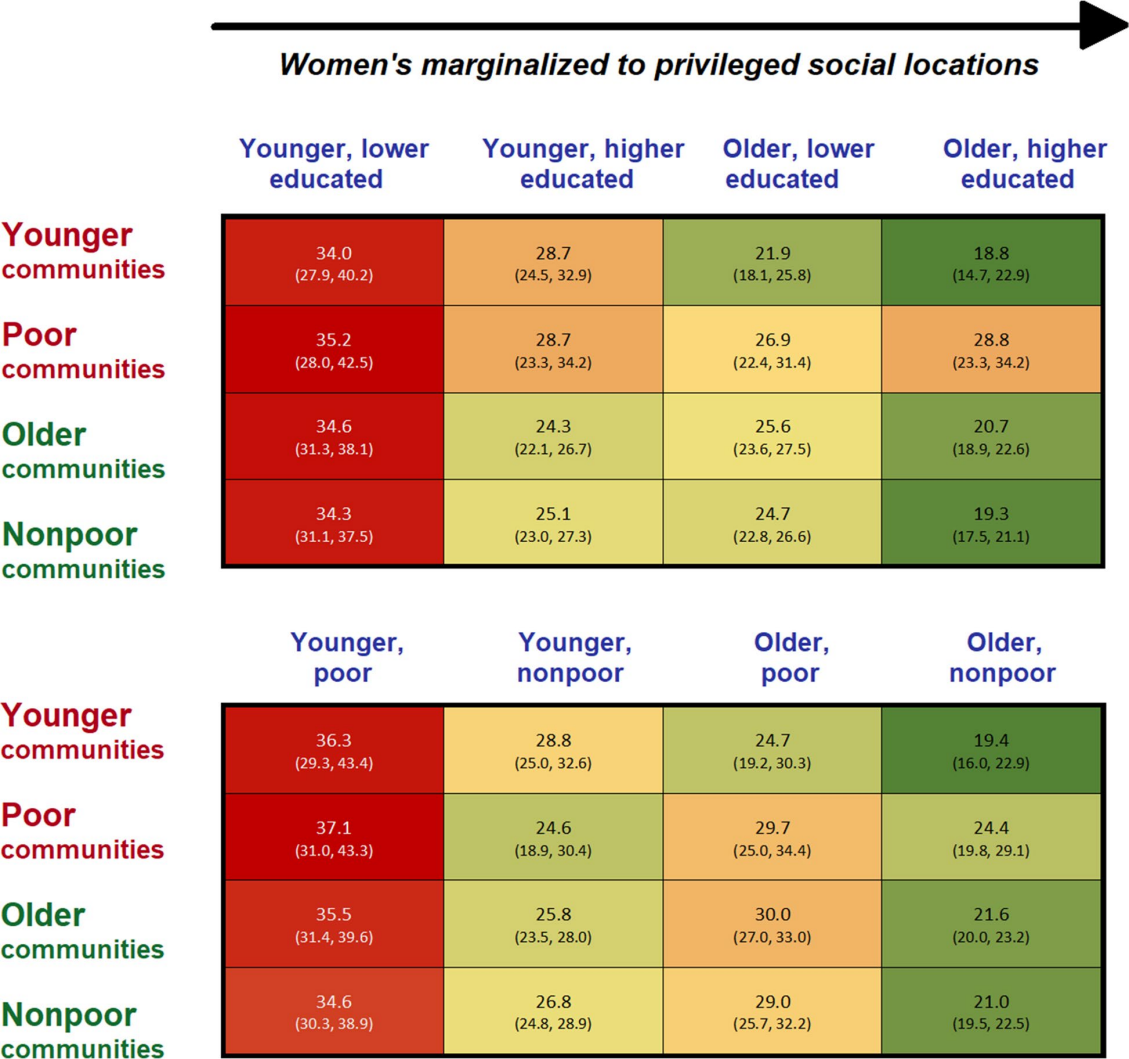
Marginal predicted probabilities (95% CI) of women experiencing physical intimate partner violence at different intersectional social locations across different types of communities.^1^Bangladesh violence against women survey 2015, unweighted N, women = 15,421; weighted N, women = 34,546,982; N, communities = 911.^2^Younger and poor communities were considered disadvantaged communities, and older and nonpoor communities, advantaged communities.^3^Dark red and green colours point to the highest and lowest predicted probabilities, the lighter shades lie in between these extreme point estimates.^4^Probabilities were estimated after running Model-2 (see Additional file 6)

Women’s intersectional social locations revealed significant differences in IPV prevalence rates, the highest for *younger, poor* women (35.4%, n_w_ = 2,735,813), the lowest for *older, higher educated* women (20.1%, n_w_ = 9,758,881; see Additional file 5). Figure 2 depicts how women’s marginal predicted probabilities of IPV at their different intersectional social locations varied within each type of community. IPV probabilities varied from 18.8% (95% CI, 14.7, 22.9) for *older, higher educated* women in younger communities to 37.1% (95% CI, 31.0, 43.3) for *younger, poor women* in poor communities (Fig. 2). *Younger, lower educated* or *poor* women’s predicted probabilities of IPV ranged from 34.0% (95% CI, 27.9, 40.2) in younger communities to 37.1% (95% CI, 31.0, 43.3) in poor communities.

### Results of testing hypothesis 1

Within community differences indicated that *younger, poor* compared to *older, nonpoor* women had significantly higher IPV probabilities (the minimum percentage point difference = 12.7, 95% CI, 2.8, 22.6) in all communities (see Additional file 7, Fig. 3). Similar trend was observed between *younger, lower educated* compared to *older, higher educated* women in all except communities that were poor. The minimum percentage point difference in IPV probabilities was marked in older communities (13.9, 95% CI, 8.2, 19.6) while the maximum, in younger communities (15.2, 95% CI, 5.0, 25.5; see Additional file 7, Fig. 3). In contrast, in poor communities, *younger, lower educated* compared to *older, higher educated* women had 6.4% (95% CI, − 7.0, 19.9) higher IPV probabilities, but this difference was not significant (see Additional file 7, Fig. 3).

**Fig. 3:**
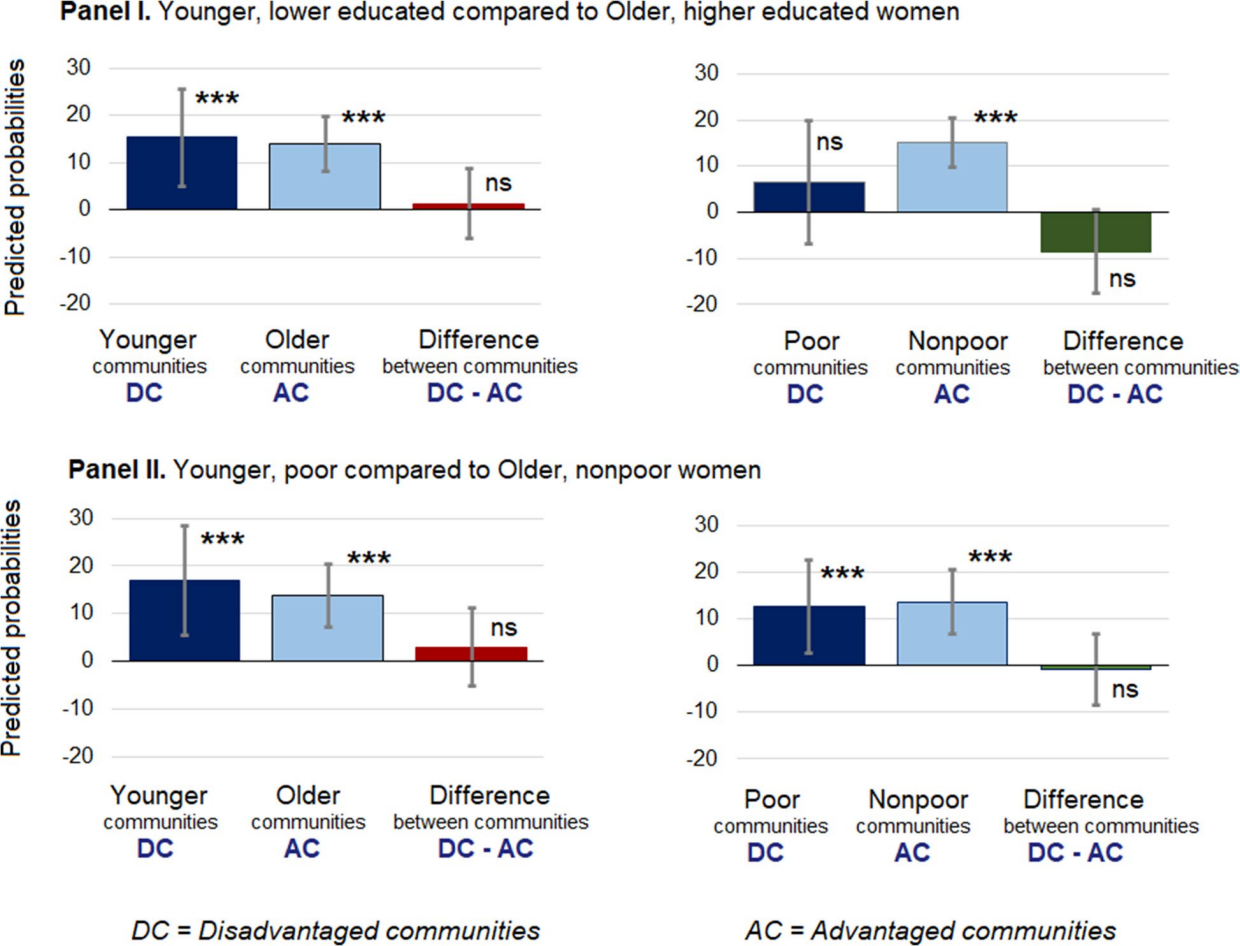
Results of testing Hypothesis 1. Within community differences and between community difference-in-differences in marginal predicted probabilities of women experiencing physical intimate partner violence between disadvantaged and advantaged communities. ***p < .000; **p < .00; * < 05; ns-nonsignificant at p < .05. DC=Disadvantaged communities; AC = Advantaged communities.^1^Bangladesh violence against women survey 2015, unweighted N, women = 15,421; weighted N, women = 34,546,982; N, communities = 911. ^2^In Panel **I**, difference-in-differences between younger and older communities = 1.3, 95% CI (− 6.1, 8.7); between poor and nonpoor communities = − 8.6, 95% CI (− 17.6, 0.5). See Additional file 7. ^3^In Panel **II**, difference-in-differences between younger and older communities = 3, 95% CI (− 5.2, 11.1); between poor and nonpoor communities = − 0.9, 95% CI (− 8.5, 6.7). See Additional file 7

The between community, difference-in-difference tests indicated no difference between disadvantaged and advantaged communities (probabilities ranged from − 8.6% (95% CI, − 17.6, 0.5) to 3.0% (95% CI, − 5.2, 11.1; see Additional file 7, Fig. 3). Therefore, we could not confirm Hypothesis 1 (i.e.*, younger, lower educated or poor compared to older, higher educated or nonpoor women will have higher IPV probabilities in disadvantaged than advantaged communities*). In other words, disparities were not higher in a disadvantaged compared to an advantaged community.

### Results of testing hypothesis 2

In all advantaged communities (i.e., older and nonpoor communities), younger women who were higher educated or nonpoor compared to their lower educated or poor counterparts had significantly lower IPV probabilities (the minimum percentage point difference = 7.8, 95% CI, 0.6, 14.9; see Additional file 8, Fig. 4). In only one type of disadvantaged communities, i.e., in poor communities, *younger, nonpoor* women had significantly lower IPV probabilities compared to *younger, poor* women (difference in probabilities = 12.5, 95% CI, 1.2, 23.8; see Additional file 8, Fig. 4).

**Fig. 4:**
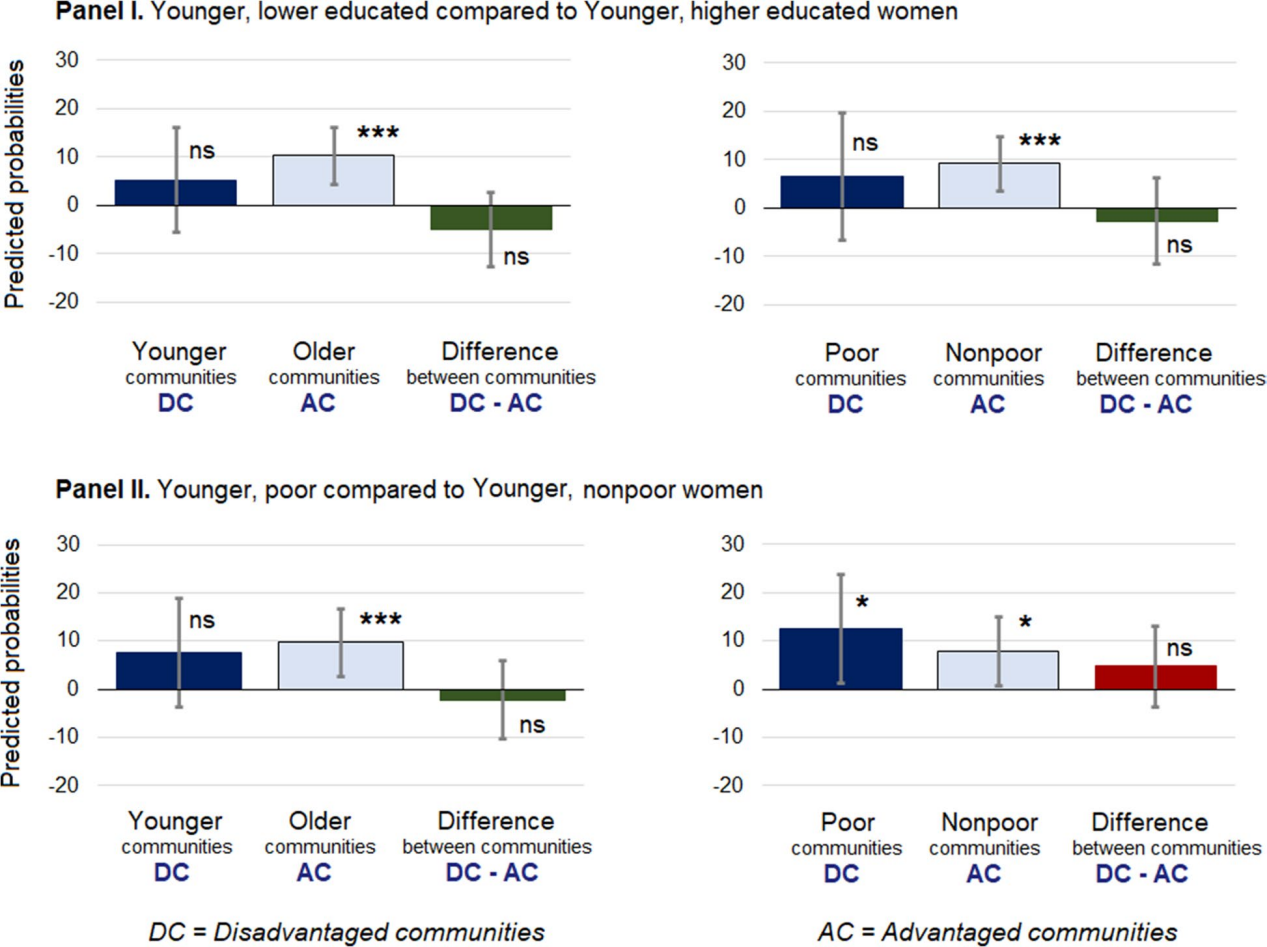
Results of testing Hypothesis 2. Within community differences and between community difference-in-differences in marginal predicted probabilities of women experiencing physical intimate partner violence between disadvantaged and advantaged communities. ***p < .000; **p < .00; * < 05; ns-nonsignificant at p < .05. DC=Disadvantaged communities; AC = Advantaged communities.^1^Bangladesh violence against women survey 2015, unweighted N, women = 15,421; weighted N, women = 34,546,982; N, communities = 911. ^2^In Panel **I**, difference-in-differences between younger and older communities = − 5.0, 95% CI (− 12.6, 2.8); between poor and nonpoor communities = − 2.7, 95% CI (− 11.7, 6.2), *p* = 0.55. See Additional file 8. ^3^In Panel **II**, difference-in-differences between younger and older communities = − 2.2, 95% CI (− 10.4, 5.9), *p* = 0.59; between poor and nonpoor communities = 4.7, 95% CI (− 3.7, 13.1), *p* = 0.27. See Additional file 8

However, there was no difference in disparities between disadvantaged and advantaged communities (difference-in-difference probabilities ranged from − 5.0, 95% CI, (− 12.6, 2.8) to 4.7, 95% CI (− 3.7, 13.1); see Additional file 8, Fig. 4). Thus, we also could not confirm Hypothesis 2 (i.e.*, younger, higher educated or nonpoor compared to younger, lower educated or poor women will have lower IPV probabilities in advantaged than disadvantaged communities*). In other words, disparities in experiencing IPV between younger, lower educated or poor and their counterparts who were higher educated or nonpoor were not more pronounced in an advantaged compared to a disadvantaged community.

### Sensitivity analyses

For hypothesis 1, sensitivity analyses (see Additional file 9) indicated significant difference between *younger, lower educated* and *older, higher educated* women in poor compared to nonpoor communities, however, this finding pointed to the opposite direction to what we had hypothesized. That is, although we assumed that the disparities will be more acute in poor compared to nonpoor communities, we found that the disparities were higher in nonpoor compared to poor communities. For Hypothesis 2, the sensitivity analyses did not find any difference between communities, confirming the findings of the primary analysis (see Additional file 10).

## Discussion

As expected, we found significant *within community differences* in experiencing IPV among younger women who were lower educated and older women who were higher educated in all communities except those that were poor. Younger women who were poor compared to older women who were nonpoor also had significantly higher probabilities of experiencing IPV in all communities. Additionally, younger, lower educated or poor women were disadvantaged compared to their counterparts who were higher educated or nonpoor in all advantaged (i.e., older and nonpoor) communities. Such widespread *within community differences* indicated that women’s age, education, and poverty are sites of both oppressions and privileges with underlying patriarchal and socio-economic power structures in Bangladesh, as elsewhere [57].

However, *within community differences* among younger women who were lower versus higher educated was not found in younger and poor communities. Most importantly, we did not find support for our hypotheses on finding *between community differences* to point to the interacting systems of power, oppressions, and privileges working together in such a way so that younger women’s probabilities of experiencing IPV are exacerbated in disadvantaged communities, while they are reduced in advantaged communities. Contrary to what we had hypothesized, poor communities revealed absence of differences in probability of women experiencing IPV at the intersection of age and education, but not at the intersection of age and poverty.

### Disadvantaged communities’ role in exacerbating disadvantages

Unexpectedly, we did not find support for Hypothesis 1, i.e., *younger, lower educated or poor* women’s IPV probabilities did not exacerbated in disadvantaged compared to advantaged communities. As expected, *younger, lower educated* women compared to *older, higher educated* women had higher probabilities of experiencing IPV in most communities, confirming the findings of an earlier study, although the authors did not examine variation across communities [14]. However, in poor communities, the hypothetically privileged, *older, higher educated* women did not occupy an advantaged location, rendering no difference between poor and nonpoor communities. This absence of difference in IPV probabilities between these marginalized and privileged groups in poor communities occurred not because *younger, lower educated* women had low levels of IPV probabilities, but the *older, higher educated* women had high levels of IPV compared to their counterparts in nonpoor communities. Therefore, such equality in poverty, as previously shown in the Canadian context (Armstrong, 1996 as cited in [58]), is not ideal. To clarify, an intersectional social justice project does not expect the oppression against a privileged group to increase to make them equally vulnerable to an oppressed group. Instead, the purpose is to reduce oppression for all groups with greater attention to those at more disadvantaged locations at an accelerated rate [59].

In contrast, the significant difference between *younger, poor* and *older, nonpoor* women in poor communities indicates the importance of material resources over education in these settings. According to gender equity and IPV literature, education that increases personal capability, skills, and employment opportunity is pivotal to empower women, and women’s empowerment is key to combating IPV [7, 40, 60]. Theoretically, education may also equip women with higher bargaining power and greater ability to resolve conflict, in turn, motivating them not to accept violence either as a patriarchal norm or an instrument of conflict resolution [15, 40, 61]. Considering this, one would expect educated women to achieve a higher social status and standard of living and be more equipped to manage stress and conflict with a concomitant reduction in experiencing IPV [14, 40]. In this study, although the social processes were not examined, it is plausible that the protective effect of education played a positive role for older and higher educated women in nonpoor communities, but not in poor communities. This might be due to the status inconsistency between spouses in education while living in a resource poor setting [62]. For example, women with higher education than husbands were more likely to experience IPV in India [46] and in a factory-based violence prevention study in Bangladesh [63]. However, studies using nationally representative data in Bangladesh found it to be either a protective factor or to have no effect although these authors did not account for poor community settings [7, 64].

### Advantaged communities’ role in ameliorating advantages

Hypothesis 2 was supported by the finding that younger women who were equipped with higher education and material resources had reduced IPV probabilities compared to younger, lower educated or poor women in advantaged (i.e., nonpoor and older) communities. Although this provided support for our underlying assumption that interacting systems of power and privileges at the community-level work together, the differences did not vary significantly between advantaged and disadvantaged communities. Interestingly, in poor communities, *younger, nonpoor* women were better off compared to *younger, poor* women; however, younger women with either lower or higher education were equally disadvantaged. This suggests that, in these communities, women’s economic status played a protective role while their levels of education did not. Indeed, an anthropological study conducted in Bangladeshi villages revealed that access to education created younger women’s ability to work, improving their family income, consequently, contributing to a decline in IPV in poor communities [61]. It is, therefore, plausible that the promise of education falls flat when it does not lead, at the same time, to a reduction in poverty; even with a higher education, poverty related stress and conflicts may lead to higher rates of IPV [14, 61].

Interestingly, in patriarchy dominated younger communities, younger women who were either higher educated or nonpoor did not get any advantage over their marginalized counterparts who were lower educated or poor, reflecting the durability of patriarchal settings that put younger women at the most precarious location irrespective of their education and economic status. In contrast, a cross-cultural study using data from 44 countries indicated that women’s education was likely to reduce violence in countries where wife abuse was normative than where it was not [65]. However, a recent study [18] in Bangladesh found that the advantage of women getting married at a later age is reversed in patriarchal communities, where high proportions of girls are married before age 15. Another study found that women’s empowerment, although a protective factor for IPV in a socially progressive setting in Bangladesh, became a risk factor in a socially conservative setting [19].

### Implications of younger women’s intersectional locations across community types

As we did not find evidence that disadvantaged communities exacerbate and advantaged communities ameliorate IPV for younger women, we cannot suggest prioritizing disadvantaged over advantaged communities for intervention. Younger, lower educated or poor women were clearly located on the bottom rung, bearing the brunt of IPV in all communities. The robust within community differences suggest that disrupting patriarchal norms and practices that put younger women at the most precarious social location and ameliorating their material and educational conditions are to be prioritized in all communities. Also, absence of difference between *younger, lower educated* and *younger or older, higher educated* women (i.e., a flattening effect of education regardless of age) might point to the problem of education not being translated into social and material benefits in poor communities [66]. Therefore, Bangladeshi IPV prevention programs and policies should invest in providing quality education and improving women’s material conditions and gender equitable relationships and norms, addressing power imbalances in all communities. A recent study found that providing educational support, skills, and gender awareness training to adolescent girls helped reducing child marriage in Bangladeshi villages [67]. Similar initiatives can be made to reduce IPV. Following the proportionate universalism approach [59] reaching out to all women in all communities, but investing more resources for younger women and communities that are poor might be justified. Future studies may examine the socio-economic-political structures and processes that underlie the power, oppressions, and privileges to reduce inequality and IPV against not just younger women, but all women. This would allow avoiding oppression Olympics or competition among multiple marginalized groups [68] towards building intersectional solidarity and effect change.

### Strengths and limitations

Embedded in intersectionality theory is the social justice project of addressing violence against women [11, 69], yet, thus far, only 1% applied quantitative intersectionality studies have incorporated this theory to examine IPV (authors’ estimate from Bauer et al.’s [70] review article). Interestingly, none of these IPV studies used or tested intersectionality theory informed hypotheses. The current study, thus, helps advancing the IPV and intercategorical intersectionality scholarship by generating and testing intersectionality theory oriented, directional hypotheses. Although intersectionality is not a testable theory [35], we drew on IPV literature to generate hypotheses. We pragmatically used existing social categories that are sites of power, oppression, and privilege, as required in intersectionality research [12]. As such, it answers recent calls to conduct intercategorical research that “embed an explicit theoretical frame to best meet the tenets of intersectionality” ( [71] p262). This study benefits from using a large sample, with a low rate of non-response (BBS, [38]). It is important to note, however, that identifying social categories with IPV or sensitive outcomes has a stigmatizing potential that might further harm [52]. On the other hand, the fear of stigmatization might also keep IPV underground [39]. To avoid this, the current study focussed on women’s social locations, not identities [32].

This study has several limitations. As it was quantitative, we could not incorporate women’s subjective experiences. Using a cross-sectional survey data allowed us only to indicate association, not causation. Due to unavailability of data, we could neither include variables on structures that generate women’s social locations, nor could we include process indicators that lead to IPV, although these are key aspects of intersectionality theory. To manage complexity, we dichotomized the variables, however, we followed previous literature to set cut-offs and conducted sensitivity analyses. Although the modified conflict tactics scale is considered a gold standard measure [1], IPV can be expressed differently across cultures [72]. Interestingly, BBS [38] included country specific items to address this issue. As BBS did not over sample some marginalized populations, we also could not include Indigenousness, disability, and gender identity categories that are some critical social locations [69]. Given that women are subject to multiple forms of violence [38], it is important to apply this analysis to examine multi-dimensional violence against women in the future. Moreover, since intersectional inequalities change across time and place [12, 31], doing a longitudinal study could have captured how intersectionality inequalities travel across the life course. Considering the enormity of IPV problem, the BBS might consider conducting longitudinal surveys to allow examination of causal processes as well as any change in IPV going forward. As women’s getting married at a young age is not common in all countries, the concept of younger communities might be unique to countries like Bangladesh, offering limited generalizability. However, the precarities of women’s lower education, poverty, and their living in poor communities are realities in many settings; therefore, the findings of this study might be partially applicable to other low- and middle-income countries.

## Conclusion

While we found no evidence that younger women’s intersecting disadvantages got worse in disadvantaged communities and improved in advantaged communities, we found significant within community differences, revealing complex inequalities within each type of community. As the purpose of an intersectional, social justice project is to reduce the underlying oppression for all social groups with greater attention to those at more disadvantaged locations, we suggest (a) addressing patriarchal norms, practices, and the economic power structures that put younger, lower educated or poor women at most precarious social locations and (b) ameliorating their material and educational conditions in all communities. Additionally, in poor communities, the flattening effect of education for older as well as younger women are to be further examined. Future studies also need to examine the socio-economic-political forces and processes that underlie the power, oppressions, and privileges at the intersectional locations to reduce inequality and IPV against not just for younger, but all women. For generating and testing intersectionality theory-oriented directional hypotheses in IPV research, this study might advance scholarship on domestic violence and quantitative intersectionality globally.

## Supplementary Information

**Additional file 1.** Flowchart on the selection of study participants and communities.

**Additional file 2.** Multilevel logistic regression model predicting the currently married women experiencing physical intimate partner violence in Bangladesh.

**Additional file 3.** Description of sensitivity analysis.

**Additional file 4.** Women and community characteristics.

**Additional file 5.** Women’s experiences of physical intimate partner violence in the past year by women and community characteristics.

**Additional file 6.** Multilevel logistic regression model estimates, coefficients (95% CI), predicting women’s experiences of physical intimate partner violence in the past year.

**Additional file 7.** Results of testing Hypothesis 1: Within and between community differences in probabilities of women experiencing physical intimate partner violence in the past year.

**Additional file 8.** Results of testing Hypothesis 2: Within and between community differences in probabilities of women experiencing physical intimate partner violence in the past year.

**Additional file 9.** Sensitivity analysis, results of testing Hypothesis 1: Within and between community differences in marginal predicted probabilities of women experiencing physical intimate partner violence in the past year.

**Additional file 10.** Sensitivity analysis, results of testing Hypothesis 2: Within and between community differences in marginal predicted probabilities of women experiencing physical intimate partner violence in the past year.

## Data Availability

Data of the current study are subset of the Bangladesh Violence Against Women Survey 2015 dataset, which is available from the Bangladesh Bureau of Statistics at http://203.112.218.65:8008/PageSiteMap1.aspx?MenuKey = 7#.

## Abbreviations

BBS: Bangladesh Bureau of Statistics
BVAWS2015: The 2015 Bangladesh violence against women survey
IPV: Intimate partner violence

## References

[1] Devries KM, Mak JYT, García-Moreno C, Petzold M, Child JC, Falder G (2013). The global prevalence of intimate partner violence against women. Science..

[2] Johnston HB, Naved RT (2008). Spousal violence in Bangladesh: a call for a public-health response. J Health Popul Nutr.

[3] Schuler SR, Hashemi SM, Riley AP, Akhter S (1996). Credit programs, patriarchy and men’s violence against women in rural Bangladesh. Soc Sci Med.

[4] Sambisa W, Angeles G, Lance PM, Naved RT, Thornton J (2011). Prevalence and correlates of physical spousal violence against women in slum and nonslum areas of urban Bangladesh. J Interpers Violence.

[5] Islam TM, Tareque MI, Tiedt AD, Hoque N (2014). The intergenerational transmission of intimate partner violence in Bangladesh. Glob Health Action.

[6] Dalal K, Rahman F, Jansson B (2009). Wife abuse in rural Bangladesh. J Biosoc Sci.

[7] Amin S, Khan TF, Rahman L, Naved RT. Mapping violence against women in Bangladesh: A multilevel analysis of demographic and health survey data. In: Naved RT, Amin S, editors. From evidence to policy: Addressing gender-based violence against women and girls in Bangladesh, 22-51. Dhaka: BD: icddr,b; 2013.

[8] Ahmed SM (2005). Intimate partner violence against women: experiences from a woman-focused development programme in Matlab, Bangladesh. J Health Popul Nutr.

[9] Crenshaw K, Lutz H, MTH V, Supik L (2011). Postscript. Framing intersectionality: Debates on a multi-faceted concept in gender studies, the feminist imagination-Europe and beyond. Kindle.

[10] Crenshaw K (1989). Demarginalizing the intersection of race and sex: a black feminist critique of antidiscrimination doctrine, feminist theory, and antiracist politics. Univ Chic Leg Forum.

[11] Crenshaw K (1991). Mapping the margins: Intersectionality, identity politics, and violence against women of color. Stanford Law Rev.

[12] McCall L (2005). The complexity of intersectionality. Signs (Chic).

[13] Beyer K, Wallis AB, Hamberger LK (2015). Neighborhood environment and intimate partner violence: a systematic review. Trauma Violence Abuse.

[14] Rahman L, Du Mont J, O’Campo P, Einstein G. Currently married women’s present experiences of male intimate partner physical violence in Bangladesh: An intercategorical intersectional approach. Glob Public Health. 2020;15(1):121–36. 10.1080/17441692.2019.1649447 31392927

[15] Bates LM, Schuler SR, Islam F, Islam K (2004). Socioeconomic factors and processes associated with domestic violence in rural Bangladesh. Int Fam Plan Perspect.

[16] O’Campo P, Gielen AC, Faden RR, Xue X, Kass N, Wang MC (1995). Violence by male partners against women during the childbearing year: a contextual analysis. Am J Public Health.

[17] Rahman L, Du Mont J, O’Campo P, Einstein G. Intersectional community correlates of married women’s experiences of male intimate partner physical violence in Bangladesh: A cross-sectional study. J Epidemiol Community Health. 2020;74:182–9. 10.1136/jech-2019-212295 PMC6993019 31722985

[18] Yount KM, Crandall A, Cheong YF, Osypuk TL, Bates LM, Naved RT (2016). Child marriage and intimate partner violence in rural Bangladesh: a longitudinal multilevel analysis. Demography..

[19] Koenig MA, Ahmed S, Hossain MB, Mozumder ABMKA (2003). Women’s status and domestic violence in rural Bangladesh: individual- and community-level effects. Demography..

[20] VanderEnde KE, Sibley LM, Cheong YF, Naved RT, Yount KM (2015). Community economic status and intimate partner violence against women in Bangladesh: compositional or contextual effects?. Violence Against Women.

[21] Amin S, Asadullah MN, Hossain S, Wahhaj Z (2017). Eradicating child marriage in the commonwealth: is investment in girls’ education sufficient?. Round Table.

[22] Bangladesh Planning Commission (2015). Seventh five-year plan, FY2016-FY2020: Accelerating growth, empowering citizens.

[23] Dobash RE, Dobash R (1979). Violence against wives : a case against the patriarchy.

[24] Chowdhury FD (2004). The socio-cultural context of child marriage in a Bangladeshi village. Int J Soc Welf.

[25] Kandiyoti D (1988). Bargaining with patriarchy. Gend Soc.

[26] Schuler SR, Lenzi R, Badal SH, Nazneen S (2018). Men’s perspectives on women’s empowerment and intimate partner violence in rural Bangladesh. Cult Health Sex.

[27] Farrington KM, Straus MA, Hotaling GT (1980). Stress and family violence. The social causes of husband-wife violence.

[28] Wright EM, Benson ML (2010). Immigration and intimate partner violence: exploring the immigrant paradox. Soc Probl.

[29] Chavis AZ, Hill MS (2008). Integrating multiple intersecting identities: a multicultural conceptualization of the power and control wheel. Women Ther.

[30] Collins PH (2000). Black feminist thought: knowledge, consciousness, and the politics of empowerment.

[31] Hankivsky O (2012). Women’s health, men’s health, and gender and health: implications of intersectionality. Soc Sci Med.

[32] Bauer GR (2014). Incorporating intersectionality theory into population health research methodology: challenges and the potential to advance health equity. Soc Sci Med.

[33] Phillips SP, Vafaei A, Yu S, Rodrigues R, Ilinca S, Zolyomi E (2020). Systematic review of methods used to study the intersecting impact of sex and social locations on health outcomes. SSM Popul Heal.

[34] O’Campo P (2012). Are we producing the right kind of actionable evidence for the social determinants of health?. J Urban Health.

[35] Del Rio-Gonzalez AM, Holt SL, Bowleg L (2021). Powering and structuring intersectionality: beyond main and interactive associations. Res Child Adolesc Psychopathol.

[36] Hancock A-M (2007). Intersectionality as a normative and empirical paradigm. Polit Gend.

[37] Liu X (2016). Applied ordinal logistic regression using Stata: from single-level to multilevel modelling. Kindle.

[38] Bangladesh Bureau of Statistics. Bangladesh Violence Against Women Survey 2015. Dhaka, Bangladesh: Government of Bangladesh; 2016.

[39] Ellsberg M, Heise L. Researching violence against women: a practical guide for researchers and activists. Washington D.C.: World Health Organization and Program for Appropriate Technology in Health; 2005.

[40] Boyle MH, Georgiades K, Cullen J, Racine Y (2009). Community influences on intimate partner violence in India: Women’s education, attitudes towards mistreatment and standards of living. Soc Sci Med.

[41] Hox JJ, Moerbeek M, van de Schoot R, Hox JJ, Moerbeek M (2018). Multilevel analysis: Techniques and applications.

[42] Wang X, Cheng Z (2020). Cross-sectional studies: strengths, weaknesses, and recommendations. Chest..

[43] Vandenbroucke JP, von Elm E, Altman DG, Gøtzsche PC, Mulrow CD, Pocock SJ, et al. Strengthening the reporting of observational studies in Epidemiology (STROBE): explanation and elaboration. Epidemiology. 2007;18(6):805–35. 10.1097/EDE.0b013e3181577511 18049195

[44] Straus MA, Hamby SL, Boney-McCoy SUE, Sugarman DB (1996). The revised conflict tactics scales (CTS2): development and preliminary psychometric data. J Fam Issues.

[45] Ismayilova L (2015). Spousal violence in 5 transitional countries: a population-based multilevel analysis of individual and contextual factors. Am J Public Health.

[46] Ackerson LK, Kawachi I, Barbeau EM, Subramanian SV (2008). Effects of individual and proximate educational context on intimate partner violence: a population-based study of women in India. Am J Public Health.

[47] Naved RT (2008). Violence against women. In 2006 Bangladesh urban health survey.

[48] Murshid NS (2017). Men’s report of domestic violence perpetration in Bangladesh: correlates from a nationally representative survey. J Interpers Violence.

[49] Fulu E, Jewkes R, Roselli T, Garcia-Moreno C (2013). Prevalence of and factors associated with male perpetration of intimate partner violence: findings from the UN multi-country cross-sectional study on men and violence in Asia and the Pacific. Lancet Glob Health.

[50] Jaccard J (2001). Interaction effects in logistic regresssion.

[51] Kolenikov S (2010). Resampling variance estimation for complex survey data. Stata J.

[52] Wemrell M, Karlsson N, Perez Vicente R, Merlo J (2021). An intersectional analysis providing more precise information on inequities in self-rated health. Int J Equity Health.

[53] Merlo J, Wagner P, Leckie G (2019). A simple multilevel approach for analysing geographical inequalities in public health reports: the case of municipality differences in obesity. Health Place.

[54] StataCorp (2017). Stata statistical software: Release 15.

[55] StataCorp (2017). Stata survey data reference manual: Release 15.

[56] von Elm E, Altman DG, Egger M, Pocock SJ, Gøtzsche PC, Vandenbroucke JP. The strengthening the reporting of observational studies in epidemiology (STROBE) statement: Guidelines for reporting observational studies. Lancet (British Ed). 2007;370(9596):1453–7. 10.1016/S0140-6736(07)61602-X 18064739

[57] Coll CVN, Ewerling F, García-Moreno C, Hellwig F, Barros AJD (2020). Intimate partner violence in 46 low-income and middle-income countries: an appraisal of the most vulnerable groups of women using national health surveys. BMJ Glob Health.

[58] McCall L (2001). Complex inequality: gender, class, and race in the new economy (perspectives on gender). Kindle.

[59] Marmot M, Bell R (2012). Fair society, healthy lives. Public Health.

[60] Rahman M, Nakamura K, Seino K, Kizuki M (2013). Does gender inequity increase the risk of intimate partner violence among women? Evidence from a national Bangladeshi sample. PLoS One.

[61] Schuler SR, Lenzi R, Nazneen S, Bates LM (2013). Perceived decline in intimate partner violence against women in Bangladesh: qualitative evidence. Stud Fam Plan.

[62] Yick AG (2001). Feminist theory and status inconsistency theory: application to domestic violence in Chinese immigrant familes. Violence Against Women.

[63] Naved RT, Al Mamun M, Parvin K, Willan S, Gibbs A, Yu M (2018). Magnitude and correlates of intimate partner violence against female garment workers from selected factories in Bangladesh. PLoS One.

[64] Murshid NS, Akincigil A, Zippay A (2016). Microfinance participation and domestic violence in Bangladesh: results from a nationally representative survey. J Interpers Violence.

[65] Heise LL, Kotsadam A (2015). Cross-national and multilevel correlates of partner violence: an analysis of data from population-based surveys. Lancet Glob Health.

[66] Chisamya G, DeJaeghere J, Kendall N, Khan MA (2012). Gender and education for all: Progress and problems in achieving gender equity. Int J Educ Dev.

[67] Amin S, Saha JS, Ahmed JA (2018). Skills-building programs to reduce child marriage in Bangladesh: a randomized controlled trial. J Adolesc Health.

[68] Hancock A-M (2007). When multiplication doesn’t equal quick addition: examining intersectionality as a research paradigm. Perspect Polit.

[69] Kelly UA (2011). Theories of intimate partner violence: from blaming the victim to acting against injustice: Intersectionality as an analytic framework. Adv Nurs Sci.

[70] Bauer GR, Churchill SM, Mahendran M, Walwyn C, Lizotte D, Villa-Rueda AA (2021). Intersectionality in quantitative research: a systematic review of its emergence and applications of theory and methods. SSM Popul Heal.

[71] Bauer GR, Scheim AI (2019). Advancing quantitative intersectionality research methods: intracategorical and intercategorical approaches to shared and differential constructs. Soc Sci Med.

[72] Mason R, Hyman I, Berman H, Guruge S, Kanagaratnam P, Manuel L (2008). Violence is an international language: Tamil women’s perceptions of intimate partner violence. Violence Against Women.

