# Supplementary material for: Intersectional inequalities in younger women’s experiences of physical intimate partner violence across communities in Bangladesh
Source: Int J Equity Health. 2022 Jan 12;21:4. doi: 10.1186/s12939-021-01587-z (PMC8756647; doi:10.1186/s12939-021-01587-z)
Supplement: Supplementary file 1 — Additional file 1. Flowchart on the selection of study participants and communities. [file 12939_2021_1587_MOESM1_ESM.docx]

Additional file 1 Flowchart on the selection of study participants and communities.


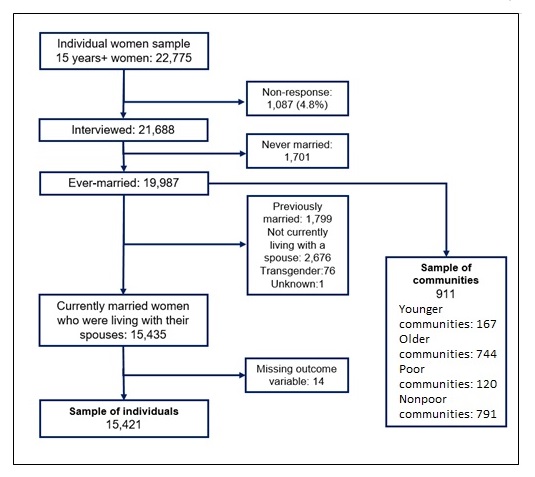


^1^Bangladesh violence against women survey 2015.

^2^Community types were generated using a larger sample of ever-married women’s (n=19987) age and poverty statuses.

^3^In community samples, younger and older communities, on the one hand, and poor and nonpoor communities, on the other hand, totalled 911 communities.
