# Supplementary material for: Intersectional inequalities in younger women’s experiences of physical intimate partner violence across communities in Bangladesh
Source: Int J Equity Health. 2022 Jan 12;21:4. doi: 10.1186/s12939-021-01587-z (PMC8756647; doi:10.1186/s12939-021-01587-z)
Supplement: Supplementary file 2 — Additional file 2. Multilevel logistic regression model predicting the currently married women experiencing physical intimate partner violence in Bangladesh. [file 12939_2021_1587_MOESM2_ESM.docx]

Additional file 2 Multilevel logistic regression model predicting the currently married women experiencing physical intimate partner violence (IPV) in Bangladesh.

logit(π_ij_)= β_0_ + β_1_ Younger age_ij_ + β_2_ Lower education_ij_ + β_3_ Poor_ij_
+ β_4_ Younger age_ij_*Lower education_ij_ + β_5_ Younger age_ij_ *Poor_ij_+ β_6_ Younger communties_j_
+ β_7_ Younger age_ij_*Younger communties_j_
+ β_8_ Lower education_ij_*Younger communties_j_+ β_9_ Poor_ij_ *Younger communties_j_+ β_10_ Younger age_ij_ *Lower education_ij_*Younger communties_j_+ β_11_ Younger age_ij_ * Poor_ij_ *Younger communties_j_+ β_12_ Poor communties_j_
+ β_13_ Younger age_ij_*Poor communties_j_ +β_14_ Lower education_ij_*Poor communties_j_+ β_15_ Poor_ij_ *Poor communties_j_+β_16_ Younger age_ij_ *Lower education_ij_*Poor communties_j_+ β_17_ Younger age_ij_ * Poor_ij_ *Poor communties_j_+ β_18_ Husband’s younger age_ij_ + β_19_ Husband’s lower education_ij_
+ β_20_ Geographical location_ij_ + β_21_ Religion_ij_ + u_j_

*Notations,* π_ij_ = Probability (IPV=1)

β_0_ = Intercept

β_1_ to β_21_ = Slopes of independent variables, interaction terms, and covariates

j = 1, …., 911 PSUs, with i = 1, …, n women in PSU_j_

u_j_ = Random effects in PSU_j_
