# Supplementary material for: Intersectional inequalities in younger women’s experiences of physical intimate partner violence across communities in Bangladesh
Source: Int J Equity Health. 2022 Jan 12;21:4. doi: 10.1186/s12939-021-01587-z (PMC8756647; doi:10.1186/s12939-021-01587-z)
Supplement: Supplementary file 3 — Additional file 3. Description of sensitivity analysis. [file 12939_2021_1587_MOESM3_ESM.docx]

Additional file 3 Description of sensitivity analysis.

Sensitivity analyses were conducted by running a multilevel logistic regression model analogous to Model 2 (Additional files 2 and 6) predicting physical intimate partner violence as in primary analysis, but in that model, level-1 binary explanatory variables were replaced with continuous variables–women’s age in years and education grades. Also, instead of using the binary variable–poor, discreet wealth quintile values were used; and communities were divided into deciles (i.e., 10 equal population groups) by community-level mean age and mean wealth quintile values.

Difference-in-differences tests were conducted in two scenarios: scenario-1, considering women’s younger age as 19 years and scenario-2, 29 years holding older age, education, poverty, and community deciles constant. In all sensitivity analyses, Decile 1 represented the younger or poor communities and Decile 9 represented the older or nonpoor communities, and older age at 69 years. Lower and higher levels of education were estimated at 4th and 11th grades education; and 1st and 5th wealth quintile households were considered poor and nonpoor women. A comparison of findings between primary and sensitivity analyses are shown in Additional files 9 and 10.
