# Supplementary material for: Intersectional inequalities in younger women’s experiences of physical intimate partner violence across communities in Bangladesh
Source: Int J Equity Health. 2022 Jan 12;21:4. doi: 10.1186/s12939-021-01587-z (PMC8756647; doi:10.1186/s12939-021-01587-z)
Supplement: Supplementary file 4 — Additional file 4. Women and community characteristics. [file 12939_2021_1587_MOESM4_ESM.docx]

Additional file 4 Women and community characteristics.

| **Particulars** | **%** | **95% CI** | **Weighted  count (n)** |
| --- | --- | --- | --- |
| **Women’s characteristics** |  |  |  |
| Younger age (15–29 years) | 34.0 | 32.9, 35.0 | 11,732,707 |
| Older age (>=30 years) | 66.0 | 65.0, 67.1 | 22,814,274 |
| Lower education (<5th grade) | 49.5 | 48.2, 50.8 | 17,108,771 |
| Higher education (>=5th grade) | 50.5 | 49.2, 51.8 | 17,438,211 |
| Poor (1st quintile) | 23.0 | 21.6, 24.5 | 7,944,569 |
| Nonpoor (2nd–5th quintiles) | 77.0 | 75.5, 78.4 | 26,602,413 |
| **Women’s intersectional locations** |  |  |  |
| Younger, lower educated | 11.7 | 11.0, 12.5 | 4,053,377 |
| Younger, higher educated | 22.3 | 21.3, 23.2 | 7,679,330 |
| Older, lower educated | 37.8 | 36.7, 38.9 | 13,055,394 |
| Older, higher educated | 28.2 | 27.1, 29.4 | 9,758,881 |
| Younger, poor | 7.9 | 7.23, 8.67 | 2,735,813 |
| Younger, nonpoor | 26.0 | 25.0, 27.1 | 8,996,895 |
| Older, poor | 15.1 | 14.0, 16.2 | 5,208,757 |
| Older, nonpoor | 51.0 | 49.6, 52.3 | 17,605,518 |
| **Community characteristics** |  |  |  |
| *Younger communities* (n=167) with high proportions of younger (<30 years) women | 17.8 | 15.1, 20.9 | 6,159,700 |
| *Older communities* (n=744) with low proportions of younger (<30 years) women | 82.2 | 79.1, 84.9 | 28,387,282 |
| *Poor communities* (n=120) with high proportions of poor (1st quintile) women | 17.1 | 14.4, 20.2 | 5,896,066 |
| *Nonpoor communities* (791) with low proportions of poor (1st quintile) women | 82.9 | 79.8, 85.6 | 28,650,916 |

^1^Bangladesh violence against women survey 2015, unweighted N, women=15,421 and weighted N, women=34,546,982; N, communities=911.

^2^Community characteristics were defined from a larger sample of 19,987 ever-married women. High-low proportion cut-offs for defining community types were 43.3% and 41.8% for younger and poor communities, respectively. For defining each community characteristic, mean plus 1 standard deviation values were used as cut-off points. Minimum, maximum, and average no. of observations per community were 8, 25, and 17, respectively.
