# Supplementary material for: Intersectional inequalities in younger women’s experiences of physical intimate partner violence across communities in Bangladesh
Source: Int J Equity Health. 2022 Jan 12;21:4. doi: 10.1186/s12939-021-01587-z (PMC8756647; doi:10.1186/s12939-021-01587-z)
Supplement: Supplementary file 5 — Additional file 5. Women’s experiences of physical intimate partner violence in the past year by women and community characteristics. [file 12939_2021_1587_MOESM5_ESM.docx]

Additional file 5 Women’s experiences of physical intimate partner violence (IPV) in the past year by women and community characteristics.

| **Variables** | **IPV-Yes** | | **IPV-No** | | **F test  (*p*)** | **Weighted  count (n)** |
| --- | --- | --- | --- | --- | --- | --- |
|  | % | 95% CI | % | 95% CI |  |  |
| All women | 25.1 | 23.7, 26.4 | 74.9 | 73.6, 76.3 |  | 34,546,982 |
| **Women’s single-axis characteristics** | | | | | | |
| Younger age (15–29 years) | 28.6 | 26.7, 30.5 | 71.4 | 69.5, 73.3 | 30.6 (0.00) | 11,732,707 |
| Older age (>=30 years) | 23.3 | 21.8, 24.8 | 76.7 | 75.2, 78.2 |  | 22,814,274 |
| Lower education  (<5th grade) | 27.8 | 26.1, 29.7 | 72.2 | 70.3, 73.9 | 31.3 (0.00) | 17,108,771 |
| Higher education  (>=5th grade) | 22.3 | 20.9, 23.8 | 77.7 | 76.2, 79.1 |  | 17,438,211 |
| Poor (1st quintile) | 31.4 | 28.9, 34.0 | 68.6 | 66.0, 71.1 | 41.9 (0.00) | 7,944,569 |
| Nonpoor (2nd-5th quintiles) | 23.2 | 21.8, 24.6 | 76.8 | 75.4, 78.2 |  | 26,602,413 |
| **Women’s intersectional social locations** | | | | | | |
| Younger, lower educated | 35.1 | 31.9, 38.4 | 64.9 | 61.6, 68.1 | 28.5  (0.00) | 4,053,377 |
| Younger, higher educated | 25.1 | 23.1, 27.2 | 74.9 | 72.8, 76.9 |  | 7,679,330 |
| Older, lower educated | 25.6 | 23.8, 27.5 | 74.4 | 72.5, 76.2 |  | 13,055,394 |
| Older, higher educated | 20.1 | 18.4, 22.0 | 79.9 | 78.0, 81.6 |  | 9,758,881 |
| Younger, poor | 35.4 | 31.7, 39.3 | 64.6 | 60.7, 68.3 | 26.7  (0.00) | 2,735,813 |
| Younger, nonpoor | 26.5 | 24.5, 28.5 | 73.5 | 71.5, 75.5 |  | 8,996,895 |
| Older, poor | 29.3 | 26.6, 32.3 | 70.7 | 67.7, 73.4 |  | 5,208,757 |
| Older, nonpoor | 21.5 | 20.0, 23.0 | 78.5 | 77.0, 80.0 |  | 17,605,518 |
| **Community characteristics** | | | | | | |
| *Younger communities* (n=167) with high proportions of younger (<30 years) women | 25.7 | 22.8, 28.8 | 74.3 | 71.2, 77.2 | 0.2  (0.66) | 6,159,700 |
| *Older communities* (n=744) with low proportions of younger  (<30 years) women | 24.9 | 23.4, 26.5 | 75.1 | 73.5, 76.6 |  | 28,387,282 |
| *Poor communities* (n=120) with high proportions of poor  (1st quintile) women | 29.5 | 25.8, 33.4 | 70.5 | 66.6, 74.2 | 7.2  (0.01) | 5,896,066 |
| *Nonpoor communities* (791) with low proportions of poor  (1st quintile) women | 24.2 | 22.8, 25.6 | 75.8 | 74.4, 77.2 |  | 28,650,916 |

^1^Bangladesh violence against women survey 2015, unweighted N, women=15,421; N, communities=911.
^2^Bi-variate associations were measured using adjusted Wald test, producing design-based F tests. ^3^F (1, 890) for women/community characteristics; F (2.98, 2652.74) and F (2.90, 2581.88) for intersections at age–education and age–poverty locations.
