# Supplementary material for: Intersectional inequalities in younger women’s experiences of physical intimate partner violence across communities in Bangladesh
Source: Int J Equity Health. 2022 Jan 12;21:4. doi: 10.1186/s12939-021-01587-z (PMC8756647; doi:10.1186/s12939-021-01587-z)
Supplement: Supplementary file 6 — Additional file 6. Multilevel logistic regression model estimates, coefficients (95% CI), predicting women’s experiences of physical intimate partner violence in the past year. [file 12939_2021_1587_MOESM6_ESM.docx]

Additional file 6 Multilevel logistic regression model estimates, coefficients (95% CI), predicting women’s experiences of physical intimate partner violence (IPV) in the past year.

|  | **Model 1** | **Model 2** |
| --- | --- | --- |
| 1. **Individual-level characteristics** |  |  |
| 1. Age |  |  |
| *Older age (>=30 years), Ref.* |  |  |
| Younger age (<30 years) |  | 0.02 (-0.17, 0.21) |
| 1. Education |  |  |
| *Higher education (<=5th grade), Ref.* |  |  |
| Lower education (<5th grade) |  | 0.06 (-0.11, 0.22) |
| 1. Poverty |  |  |
| *Nonpoor (2nd–5th quintiles), Ref.* |  |  |
| Poor (1st quintile) |  | 0.30** (0.10, 0.50) |
| 1. **Individual-level interactions** |  |  |
| 1. Younger age x Lower education |  |  |
| *Older age, higher education, Ref.* |  |  |
| Younger age, lower education |  | 0.29* (0.02, 0.55) |
| 1. Younger age x Poor |  |  |
| *Older age, nonpoor, Ref.* |  |  |
| Younger age, poor |  | -0.11 (-0.44, 0.22) |
| 1. **Community-level characteristics** |  |  |
| 1. *Older communities  (where low proportions of women were <30 years old), Ref.* |  |  |
| Younger communities  *(where high proportions of women were <30 years old)* |  | -0.11 (-0.44, 0.23) |
| 1. *Nonpoor communities  (where low proportions of poor women lived), Ref.* |  |  |
| Poor communities  *(where high proportions of poor women lived)* |  | 0.40* (0.06, 0.74) |
| 1. **Cross-level interactions** |  |  |
| 1. ***Younger age x Lower education x Younger communities*** |  |  |
| *Older age,* higher education, *older communities, Ref.* |  |  |
| Younger age, lower education, younger communities |  | -0.15 (-0.71, 0.42) |
| 1. ***Younger age x Poor x Younger communities*** |  |  |
| *Older age,* nonpoor, *older communities, Ref.* |  |  |
| Younger age, poor, younger communities |  | 0.04 (-0.51, 0.60) |
| 1. ***Younger age x Lower education x Poor communities*** |  |  |
| *Older age,* higher education, *nonpoor communities, Ref.* |  |  |
| Younger age, lower education, poor communities |  | 0.23 (-0.34, 0.80) |
| 1. ***Younger age x Poor x Poor*** ***communities*** |  |  |
| *Older age,* nonpoor, *nonpoor communities, Ref.* |  |  |
| Younger age, poor, poor communities |  | 0.43 (-0.12, 0.98) |
| **Goodness of fit and diagnostic accuracy** |  |  |
| Wald Chi-square (p) |  | 296.17 (0.00) |
| Random effects, between community variance (95% CI) | .68 (0.55, 0.83) | .65 (0.53, 0.80) |
| Area under the receiver operating  characteristic curve (AUC) in % (95% CI) | 75.66 (74.82, 76.51) | 77.05 (76.22, 77.88) |
| Change in AUC, Model-2 vs. Model-1 in % |  | 1.84 |
| Chi-square (p) |  | 23.62 (0.00) |
| Intraclass correlation coefficient (ICC) in % (95% CI) | 17.06 (14.40, 20.10) | 16.50 (13.91, 19.45) |
| Change in ICC, Model-2 vs. Model-1 in % |  | 3.32 |

**** p<0.001; ** p<0.01; * p<0.05; ~p<0.10.*

^1^Bangladesh violence against women survey 2015.

^2^Bangladesh Bureau of Statistics (BBS), Government of Bangladesh, administered the survey following the World Health Organization’s safety and ethical guidelines. It was a nationally representative, household-based survey, generating a cross-sectional, one time point dataset covering all the then seven divisions of Bangladesh. The survey used a stratified two-stage cluster survey design. Some 21,688 women and girls of 15 years or older were successfully interviewed from a sample of 22,775 women and girls, rendering a 95.2% response rate.

^163^A sub-sample of this survey comprising 15,421 currently married women, 15 years or older, who were living with their husbands during the survey across 911 primary sampling units (PSUs, representing communities in this study) in August 2015 were analysed in this study.

^4^Community characteristics were defined from a larger sample of 19,987 ever-married women. High-low proportion cut-off points for defining community types were 43.3% and 41.8% for younger and poor communities, respectively. For defining each community characteristic, mean plus 1 standard deviation values were used as cut-off points.

^5^Minimum, maximum, and average no. of observations per community were 8, 25, and 17, respectively.

^6^Model-2 additionally accounted for women’s religion, rural location, and their husband’s age and education.

^7^Mean-variance adaptive Gauss–Hermite quadrature integration method ^26^ have been used for estimation; and unstructured covariance structure estimated all variances and covariances. Individual- and community-level survey weights were applied in all models.
