# Supplementary material for: Intersectional inequalities in younger women’s experiences of physical intimate partner violence across communities in Bangladesh
Source: Int J Equity Health. 2022 Jan 12;21:4. doi: 10.1186/s12939-021-01587-z (PMC8756647; doi:10.1186/s12939-021-01587-z)
Supplement: Supplementary file 7 — Additional file 7. Results of testing Hypothesis 1: Within and between community differences in probabilities of women experiencing physical intimate partner violence in the past year. [file 12939_2021_1587_MOESM7_ESM.docx]

Additional file 7 Results of testing Hypothesis 1: Within and between community differences in probabilities of women experiencing physical intimate partner violence in the past year.

| **Community types** | Marginal predicted probabilities at women’s each intersectional social location (95% CI) | | **Within community differences**  (95% CI) | ***Between community difference-in-differences***  (95% CI) |
| --- | --- | --- | --- | --- |
| **Panel I.** Younger, lower educated and Older, higher educated women | | | | |
|  | A. Younger, lower educated | B. Older, higher educated | C (A – B) | D |
| 1. Younger communities^DC^ | 34.0  (27.9, 40.2) | 18.8  (14.7, 22.9) | 15.2  (5.0, 25.5)*** |  |
| 2. Older communities^AC^ | 34.6  (31.3, 38.1) | 20.7  (18.9, 22.6) | 13.9  (8.2, 19.6)*** |  |
| *Difference-in-Differences  (C1–C2)* | | |  | *1.3*  *(–6.1, 8.7)* |
| 3. Poor communities^DC^ | 35.2  (28.0, 42.5) | 28.8  (23.3, 34.2) | 6.4  (–7.0, 19.9) |  |
| 4. Nonpoor communities^AC^ | 34.3  (31.1, 37.5) | 19.3  (17.5, 21.1) | 15.0  (9.6, 20.3)*** |  |
| *Difference-in-Differences  (C3–C4)* | | |  | *–8.6*  *(–17.6, 0.5)* |
| **Panel II.** Younger, poor and Older, nonpoor women | | | | |
|  | E. Younger, poor | F. Older,  nonpoor | G (E – F) | H |
| 1. Younger communities^DC^ | 36.3  (29.3, 43.4) | 19.4  (16.0, 22.9) | 16.9  (5.4, 28.4)*** |  |
| 2. Older communities^AC^ | 35.5  (31.4, 39.6) | 21.6  (20.0, 23.2) | 13.9  (7.3, 20.5)*** |  |
| *Difference-in-Differences*  *(G1–G2)* | | |  | *3.0*  *(–5.2, 11.1)* |
| 3. Poor communities^DC^ | 37.1  (31.0, 43.3) | 24.4  (19.8, 29.1) | 12.7  (2.8, 22.6)*** |  |
| 4. Nonpoor communities^AC^ | 34.6  (30.3, 38.9) | 21.0  (19.5, 22.5) | 13.6  (6.7, 20.5)*** |  |
| *Difference-in-Differences*  *(G3–G4)* | | |  | *–0.9*  *(–8.5, 6.7)* |

****p<.001; **p<.01; *<05.* DC=Disadvantaged communities; AC=Advantaged communities.

^1^Bangladesh violence against women survey 2015, unweighted N, women=15,421; N, communities=911.

^2^In younger communities >43.3% married younger women lived. In poor communities, >41.8% poor married women lived.

^3^Probability estimates were calculated after running Model 2 (Additional file 6), adjusted for women’s religion, geographical location, poverty, and their husband’s age and education.

^4^The model Wald Chi-square=296.17, *p*=0.00; random effects, between community variance= 0.65, 95% CI (0.53, 0.80); and intraclass correlation coefficient =16.50, 95% CI (13.91, 19.45). Compared to a null model, this model’s area under the receiver operating characteristic curve increased significantly to 77.1% (95% CI, 76.2, 77.9), indicating a very large discriminatory accuracy.
