# Supplementary material for: Intersectional inequalities in younger women’s experiences of physical intimate partner violence across communities in Bangladesh
Source: Int J Equity Health. 2022 Jan 12;21:4. doi: 10.1186/s12939-021-01587-z (PMC8756647; doi:10.1186/s12939-021-01587-z)
Supplement: Supplementary file 8 — Additional file 8. Results of testing Hypothesis 2: Within and between community differences in probabilities of women experiencing physical intimate partner violence in the past year. [file 12939_2021_1587_MOESM8_ESM.docx]

Additional file 8 Results of testing Hypothesis 2: Within and between community differences in probabilities of women experiencing physical intimate partner violence in the past year.

| **Community types** | Marginal predicted probabilities at women’s each intersectional social location (95% CI) | | **Within community differences** (95% CI) | ***Between community difference-in-differences***  (95% CI) |
| --- | --- | --- | --- | --- |
| **Panel I.** Younger, lower educated and Younger, higher educated women | | | | |
|  | A. Younger, lower educated | B. Younger, higher educated | C (A – B) | D |
| 1. Younger communities^DC^ | 34.0  (27.9, 40.2) | 28.7  (24.5, 32.9) | 5.3  (–5.5, 16.2) |  |
| 2. Older communities^AC^ | 34.6  (31.3, 38.1) | 24.3  (22.1, 26.7) | 10.3  (4.4, 16.2)*** |  |
| *Difference-in-Differences*  *(C1–C2)* | | |  | *–5.0*  *(–12.6, 2.8)* |
| 3. Poor communities^DC^ | 35.2  (28.0, 42.5) | 28.7  (23.3, 34.2) | 6.5  (–6.7, 19.6) |  |
| 4. Nonpoor communities^AC^ | 34.3  (31.1, 37.5) | 25.1  (23.0, 27.3) | 9.2  (3.6, 14.8)*** |  |
| *Difference-in-Differences*  *(C3–C4)* | | |  | *–2.7*  *(–11.7, 6.2)* |
| **Panel II.** Younger poor and Younger nonpoor women | | | | |
|  | E. Younger, poor | F. Younger,  nonpoor | G (E – F) | H |
| 1. Younger communities^DC^ | 36.3  (29.3, 43.4) | 28.8  (25.0, 32.6) | 7.5  (–3.8, 18.8) |  |
| 2. Older communities^AC^ | 35.5  (31.4, 39.6) | 25.8  (23.5, 28.0) | 9.7  (2.8, 16.7)*** |  |
| *Difference-in-Differences*  *(G1–G2)* | | |  | *–2.2*  *(–10.4, 5.9)* |
| 3. Poor communities^DC^ | 37.1  (31.0, 43.3) | 24.6  (18.9, 30.4) | 12.5  (1.2, 23.8)* |  |
| 4. Nonpoor communities^AC^ | 34.6  (30.3, 38.9) | 26.8  (24.8, 28.9) | 7.8  (0.6, 14.9)* |  |
| *Difference-in-Differences*  *(G3–G4)* | | |  | *4.7*  *(–3.7, 13.1)* |

****p<.001; **p<.01; *<05.* DC=Disadvantaged communities; AC=Advantaged communities.

^1^Bangladesh violence against women survey 2015, unweighted N, women=15,421; N, communities=911.

^2^In younger communities >43.3% married younger women lived. In poor communities, >41.8% poor married women lived.

^3^Probability estimates were calculated after running Model 2 (Additional file 6), adjusted for women’s religion, geographical location, poverty, and their husband’s age and education.

^4^The model Wald Chi-square=296.17, *p*=0.00; random effects, between community variance= 0.65, 95% CI (0.53, 0.80); and intraclass correlation coefficient =16.50, 95% CI (13.91, 19.45). Compared to a null model, this model’s area under the receiver operating characteristic curve increased significantly to 77.1% (95% CI (76.2, 77.9)), indicating a very large discriminatory accuracy.
