# Supplementary material for: Intersectional inequalities in younger women’s experiences of physical intimate partner violence across communities in Bangladesh
Source: Int J Equity Health. 2022 Jan 12;21:4. doi: 10.1186/s12939-021-01587-z (PMC8756647; doi:10.1186/s12939-021-01587-z)
Supplement: Supplementary file 9 — Additional file 9. Sensitivity analysis, results of testing Hypothesis 1: Within and between community differences in marginal predicted probabilities of women experiencing physical intimate partner violence in the past year. [file 12939_2021_1587_MOESM9_ESM.docx]

Additional file 9 Sensitivity analysis, results of testing Hypothesis 1: Within and between community differences in marginal predicted probabilities of women experiencing physical intimate partner violence in the past year.

| **Comparisons** | **Difference-in-differences probabilities** | **95% CI** | **z** | **p>\| z \|** |
| --- | --- | --- | --- | --- |
| ***I. Younger, lower educated vs. older, higher educated women*** | | | | |
| *A. Primary analysis* |  |  |  |  |
| Younger vs. Older communities | 1.3 | *–6.1, 8.7* | 0.4 | 0.73 |
| Poor vs. Nonpoor communities | –8.6 | –17.6, 0.5 | –1.9 | 0.06 |
| *B. Sensitivity analysis, Scenario-1* | | | | |
| Younger vs. Older communities | 14.8 | –1.6, 31.3 | 1.8 | 0.08 |
| Poor vs. Nonpoor communities | –22.5 | –40.1, –4.9 | **–2.5** | **0.01** |
| *C. Sensitivity analysis, Scenario-2* | | | | |
| Younger vs. Older communities | 10.3 | –4.8, 25.4 | 1.3 | 0.18 |
| Poor vs. Nonpoor communities | –22.1 | –38.0, –6.1 | **–2.7** | **0.01** |
| ***II. Younger, poor vs. older, nonpoor women*** | | | | |
| *A. Primary analysis* |  |  |  |  |
| Younger vs. Older communities | 3.0 | –5.2, 11.1 | 0.7 | 0.47 |
| Poor vs. Nonpoor communities | –0.9 | –8.5, 6.7 | –0.2 | 0.81 |
| *B. Sensitivity analysis, Scenario-1* | | | | |
| Younger vs. Older communities | 14.7 | 3.0, 26.5 | **2.5** | **0.01** |
| Poor vs. Nonpoor communities | –6.1 | –20.5, 8.3 | –0.8 | 0.40 |
| *C. Sensitivity analysis, Scenario-2* | | | | |
| Younger vs. Older communities | 7.8 | –3.7, 19.4 | 1.3 | 0.19 |
| Poor vs. Nonpoor communities | –5.2 | –19.1, 8.7 | –0.7 | 0.47 |

^1^In primary analysis, less than 30 years old women with 4th grade or lower levels of education were considered younger, lower educated; and greater than 30 years old women belonging to 1st wealth quintile households were considered younger, poor women. However, in all sensitivity analyses, Decile 1 represented the younger or poor communities and Decile 9 represented the older or nonpoor communities.

^2^In Panel-I, Scenario-1, 19 years old women with 4th grade education were compared with 69 years old with 11th grade education. In Panel-I, Scenario-2, only the younger women’s age was considered 29 while other values used in Scenario-1 remained constant.

^3^In Panel-II, Scenario-1, 19 years old women belonging to 1st wealth quintile households were compared with 69 years old women belonging to 5th wealth quintile households. In Panel-II, Scenario-2, only the younger women’s age was considered 29 while other values used in Scenario-1 remained constant.
