# Supplementary material for: Intersectional inequalities in younger women’s experiences of physical intimate partner violence across communities in Bangladesh
Source: Int J Equity Health. 2022 Jan 12;21:4. doi: 10.1186/s12939-021-01587-z (PMC8756647; doi:10.1186/s12939-021-01587-z)
Supplement: Supplementary file 10 — Additional file 10. Sensitivity analysis, results of testing Hypothesis 2: Within and between community differences in marginal predicted probabilities of women experiencing physical intimate partner violence in the past year. [file 12939_2021_1587_MOESM10_ESM.docx]

**Additional file 10** Sensitivity analysis, results of testing Hypothesis 2: Within and between community differences in marginal predicted probabilities of women experiencing physical intimate partner violence in the past year.

| **Comparisons** | **Difference-in-differences probabilities** | **95% CI** | **z** | **p>\| z \|** |
| --- | --- | --- | --- | --- |
| ***I. Younger, lower educated vs. younger, higher educated women*** | | | | |
| *A. Primary analysis* |  |  |  |  |
| Younger vs. Older communities | –5.0 | *–12.6, 2.8* | –1.25 | 0.21 |
| Poor vs. Nonpoor communities | –2.7 | –11.7, 6.2 | –0.6 | 0.55 |
| *B. Sensitivity analysis, Scenario-1* | | | | |
| Younger vs. Older communities | 0.8 | –6.9, 8.4 | 0.2 | 0.85 |
| Poor vs. Nonpoor communities | 0.2 | –7.5, 7.9 | 0.1 | 0.96 |
| *C. Sensitivity analysis, Scenario-2* | | | | |
| Younger vs. Older communities | 2.3 | –3.1, 7.8 | 0.8 | 0.40 |
| Poor vs. Nonpoor communities | 4.1 | –1.4, 9.5 | 1.5 | 0.14 |
| ***II. Younger, poor vs. younger, nonpoor women*** | | | | |
| *A. Primary analysis* |  |  |  |  |
| Younger vs. Older communities | –2.2 | –10.4, 5.9 | –0.54 | 0.59 |
| Poor vs. Nonpoor communities | 4.7 | –3.7, 13.1 | 1.1 | 0.27 |
| *B. Sensitivity analysis, Scenario-1* | | | | |
| Younger vs. Older communities | –9.0 | –22.5, 4.5 | –1.3 | 0.19 |
| Poor vs. Nonpoor communities | 5.9 | –8.6, 20.5 | 0.8 | 0.43 |
| *C. Sensitivity analysis, Scenario-2* | | | | |
| Younger vs. Older communities | –3.6 | –12.8, 5.5 | –0.8 | 0.44 |
| Poor vs. Nonpoor communities | 4.8 | –5.2, 14.7 | 0.9 | 0.35 |

^1^In primary analysis, less than 30 years old women with 4th grade or lower levels of education were considered younger, lower educated; and greater than 30 years old women belonging to 1st wealth quintile households were considered younger, poor women. However, in all sensitivity analyses, Decile 1 represented the younger or poor communities and Decile 9 represented the older or nonpoor communities.

^2^In Panel-I, Scenario-1, 19 years old women with 4th grade education were compared with 19 years old with 11th grade education. In Panel-I, Scenario-2, only the younger women’s age was considered 29 while other values used in Scenario-1 remained constant.

^3^In Panel-II, Scenario-1, 19 years old women belonging to 1st wealth quintile households were compared with 19 years old women belonging to 5th wealth quintile households. In Panel-II, Scenario-2, only the younger women’s age was considered 29 while other values used in Scenario-1 remained constant.
